# Unilaterales Sicca-Syndrom

**DOI:** 10.1007/s00347-020-01168-5

**Published:** 2020-07-09

**Authors:** J. Jakob-Girbig, D. Meller

**Affiliations:** Universitätsklinikum Jena, Am Klinikum 1, 07747 Jena, Deutschland

**Keywords:** Keratitis superficialis punctata, Contusio bulbi, Stufendiagnostik, MRT, Tränendrüsenatrophie, Superficial punctate keratitis, Contusion of the bulb, Stepwise diagnostic procedure, Magnetic resonance imaging, Lacrimal gland atrophy

## Abstract

Es wird über den Fall eines streng einseitigen Sicca-Syndroms bei einem männlichen Patienten berichtet. Im Rahmen einer Stufendiagnostik konnte das Spektrum möglicher Ursachen immer weiter eingegrenzt werden, wobei v. a. die MRT-morphologische Darstellung der Tränendrüsenloge auf der betroffenen Seite wichtige diagnostische Informationen lieferte. Schlussendlich konnte in Zusammenschau der Befunde und in Verbindung mit der Anamnese des Patienten eine traumatisch bedingte Atrophie der Tränendrüse als auslösender Faktor eruiert werden.

## Abstract (en)

This article presents the case of a strictly unilateral dry eye syndrome in a male patient. Based on a stepwise diagnostic procedure the spectrum of possible causes could be gradually limited, whereby the magnetic resonance imaging of the lacrimal gland in particular provided important diagnostic information. Ultimately, in the synopsis of the findings and combined with the medical history of the patient, a traumatic atrophy of the lacrimal gland could be determined as the triggering factor.

## Falldarstellung

Hinter dem Sicca-Syndrom können sich die unterschiedlichsten Genesen verbergen. Gerade dann, wenn eine eher atypische Form des Krankheitsbildes vorliegt, ist es von besonderer Bedeutung durch ein stringentes diagnostisches Vorgehen deren Ursachen möglichst umfassend abzuklären.

### Anamnese

Ein 38-jähriger Patient klagte über ein seit Längerem bestehendes Druckgefühl, Brennen, Stechen sowie eine Abnahme der Sehschärfe am rechten Auge.

Er gab an, bei Verdacht auf eine Konjunktivitis bereits mit Ofloxacin- und Dexamethason-Augentropfen behandelt worden zu sein, ohne dass eine Besserung eingetreten wäre.

Zur Vorgeschichte berichtete der Patient, in seinem 20. Lebensjahr eine schwere Contusio bulbi mit Lidverletzung am rechten Auge erlitten zu haben, die operativ versorgt wurde.

In der Eigenanamnese bekannt war eine milde Rosazea ohne Notwendigkeit diesbezüglicher therapeutischer Maßnahmen.

Vor allem bei der Ausübung seiner beruflichen Tätigkeit als Tiefbauer sei er durch das unangenehme Gefühl an seinem rechten Auge sehr gestört. Am linken Auge gebe es keine Schwierigkeiten.

### Befund

Es konnte am rechten Auge ein Visus von 0,5 LogMAR und am linken Auge von 0,0 LogMAR ermittelt werden. Der Augeninnendruck war mit 18 mm Hg rechts und 17 mm Hg links normwertig. Spaltlampenmikroskopisch zeigte sich am rechten Auge eine kleine Erosio corneae bei ausgeprägter Keratitis superficialis punctata. Die Hornhaut links stellte sich glatt, klar und spiegelnd dar. Beidseits lag eine diskrete Meibom-Drüsen-Dysfunktion mit geringgradiger Blepharitis posterior vor bei intraokularer Reizfreiheit, spielender Pupille und altersentsprechendem Linsenbefund. Auffällig war der am rechten Auge nicht messbare Tränenmeniskus, während am linken Auge eine Meniskushöhe von ca. 0,2 mm ermittelt werden konnte. Fundoskopisch zeigten sich beidseits eine randscharfe, vitale Papille, eine trockene Makula mit Wallreflex und eine zirkulär anliegende Netzhaut.

### Diagnose

Break-up-Time und Schirmer-II-Test stellten sich rechts mit 3 s und 0 mm deutlich pathologisch dar. Am linken Auge konnte eine Break-up-Time von 10 s und ein Schirmer-II-Test von 15 mm ermittelt werden.

Zum Ausschluss asymmetrisch beginnender systemischer Erkrankungen, die eine Hyposekretion der Tränendrüse auslösen können, wurden serologische Testungen vorgenommen. So wurden SS-A- und SS-B-Antikörper (Sjögren-Syndrom) sowie Anti-BP-180 und Anti-BP-230 (Pemphigoid) bestimmt, die sich als negativ erwiesen. Zusätzlich konnte bei im Normbereich befindlichem löslichem Interleukin-2-Rezeptor und Angiotensin-Converting-Enzym die Manifestation einer Sarkoidose im Bereich der Tränendrüse ausgeschlossen werden.

**Figure 1:**
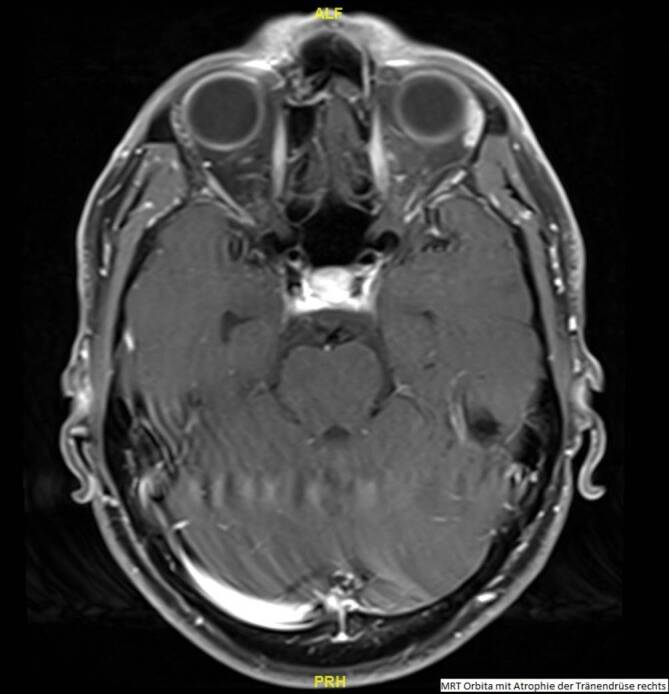

Aufgrund der vollständig aufgehobenen Sekretionsleistung der Tränendrüse rechts wurde ein MRT der Orbita nativ und mit Kontrastmittel durchgeführt. Hierbei zeigte sich eine kaum abgrenzbare Tränendrüse rechts, deren Parenchym fettig atrophiert wirkte, bei unauffällig darstellbarer Tränendrüse links (Abb. 1).

Zum Ausschluss entzündlicher oder neoplastischer Prozesse der rechten Tränendrüse erfolgte eine Biopsie. Hierbei konnten keine Hinweise für eine granulomatöse Entzündung oder ein malignes Geschehen gefunden werden.

### Therapie und Verlauf

Bei streng einseitiger totaler Hyposekretion mit MRT-morphologisch gesicherter Atrophie und bioptischem Ausschluss entzündlicher oder maligner Prozesse der Tränendrüse rechts nach Ausschluss systemischer Erkrankungen und Zustand nach schwerer Contusio bulbi mit Lidverletzung vor ca. 18 Jahren ergab sich die Diagnose einer traumatisch bedingten Atrophie der Tränendrüse rechts.

Es wurde eine Therapie mit konservierungsmittel- und phosphatfreien Tränenersatzmitteln eingeleitet und Punctum Plugs am rechten Auge eingesetzt. Zur Besserung der Funktion der ebenfalls an der Tränenfilmproduktion beteiligten Lidranddrüsen wurde bei diskreter Blepharitis posterior eine orale Therapie mit Doxycyclin Tabl. 100 mg täglich für 6 Wochen eingeleitet und dem Patienten zusätzlich Lidrandhygiene empfohlen. Zur Minderung der entzündlichen Komponente des Sicca-Syndroms wurde eine Therapie mit lokalem Ciclosporin A (Ikervis®, Santen GmbH, München, Deutschland) begonnen. Unter der genannten Therapie kam es zu einer kurzfristigen Stabilisierung des Befundes am rechten Auge mit Verschluss der Erosio corneae. Allerdings zeigte sich nur kurze Zeit später eine erneute Erosio bei wieder deutlich ausgeprägter Keratitis superficialis punctata. Aus diesem Grund wurde zur Therapieerweiterung ein Antrag bei der betreuenden Krankenkasse auf Kostenübernahme einer Therapie mit autologen Serumaugentropfen gestellt. Der Patient klagte zunehmend darüber, dass durch seine Tätigkeit als Tiefbauer mit hoher Staub- und Schmutzbelastung die Reizung seines rechten Auges verschlimmert würde. Auch die regelmäßige Applikation der notwendigen Lokaltherapie sei im Rahmen seiner beruflichen Tätigkeit kaum suffizient möglich. Zur diesbezüglichen Unterstützung wurde eine sozialdienstliche Beratung des Patienten zur Erörterung einer möglichen beruflichen Umorientierung veranlasst.

## Diskussion

Das Sicca-Syndrom wird in 2 Hauptgruppen unterteilt: hyposekretorisch und evaporativ. Die Tränendrüse stellt dabei die ursächliche Struktur der hyposekretorischen Form dar, die weiterhin untergliedert wird in Sjögren- und Nicht-Sjögren-Syndrom [1]. Das nicht-Sjögren-bedingte hyposekretorische Sicca-Syndrom resultiert dabei entweder aus einer Störung der Sekretionssteuerung der Tränendrüse, z. B. durch Schädigung der entsprechenden Afferenzen, aus einem Verschluss der Drüsenausführungsgänge oder aus einer Schädigung des Drüsengewebes [1]. Eine Schädigung des Drüsengewebes mit nachfolgender Atrophie findet sich im Rahmen des normalen Alterungsprozesses [2], als Auswirkung ionisierender Strahlung [3], als Resultat einer Dysregulation der Sexualhormone [4], bei Vorliegen einer Graft-versus-host-Disease oder nach einem Trauma [1], wobei nur im Falle einer Destruktion durch ionisierende Strahlung oder eines Traumas ein unilateraler Prozess möglich ist [5]. Die Atrophie lässt sich dabei am besten mittels Bildgebung in Form eines MRT-Scans nachweisen [5].

Im vorliegenden Fall wurde anhand einer Stufendiagnostik zuerst die hyposekretorische (und in Mischform vorliegende evaporative) Form des Sicca-Syndroms mit strenger Einseitigkeit ermittelt. Anschließend wurde mittels Labordiagnostik eine evtl. asymmetrisch beginnende systemische Erkrankung unter Einschluss der Tränendrüse ausgeschlossen. Durch die MRT-Bildgebung konnte schlussendlich die Atrophie der Tränendrüse nachgewiesen und via Biopsie eine lokale entzündliche Genese negiert werden. Somit stellten sich als mögliche Ursachen des streng einseitigen Sicca-Syndroms nur noch eine Schädigung durch ionisierende Strahlung oder ein Trauma heraus. Aufgrund der anamnestisch stattgehabten schweren Contusio bulbi mit Lidverletzung rechts konnte die Diagnose eines unilateralen hyposekretorischen Sicca-Syndroms bei traumatisch bedingter Atrophie der Tränendrüse gestellt werden.

Die eingeleitete Therapie umfasste in erster Linie Maßnahmen zur Stabilisierung der Benetzungssituation der Augenoberfläche und Minderung entzündlicher Komponenten des Sicca-Syndroms. Aufgrund der Notwendigkeit einer sehr intensiven Therapie und der ungünstigen beruflichen Voraussetzungen müssen im Rahmen eines umfassenden therapeutischen Konzeptes im vorliegenden Fall auch Maßnahmen der beruflichen Neuorientierung erörtert werden.

In der hier vorgestellten Kasuistik wird die Bedeutung einer suffizienten Stufendiagnostik zur Evaluation der Genese eines Krankheitsbildes hervorgehoben. In Verbindung mit einer ausführlichen anamnestischen Erhebung konnte in diesem Fall die seltene Genese eines Sicca-Syndroms durch traumatisch bedingte Tränendrüsenatrophie herausgearbeitet werden.

## Fazit für die Praxis

- Das Sicca-Syndrom stellt ein Krankheitsbild mit einem breiten Spektrum an möglichen Ursachen dar.
- Oftmals können diese nur eher unspezifisch bzw. unzureichend erörtert werden.
- Trotzdem sollte die diesbezügliche Abklärung so strukturiert und umfassend wie möglich erfolgen, um auch eher seltene Entitäten zu erfassen und dementsprechend zielgerichtet behandeln zu können
